# FLAIR Hyperintense Vessel Sign of Both MCAs with Severe Heart Failure

**DOI:** 10.1155/2016/5169056

**Published:** 2016-08-25

**Authors:** Donghee Kim, Seung-Yul Lee, Kwon-Duk Seo

**Affiliations:** ^1^Department of Neurology, Wonkwang University Sanbon Hospital, Sanbonro 321, Gunpo, Gyeonggi-do 15-865, Republic of Korea; ^2^Department of Cardiology, Wonkwang University Sanbon Hospital, Sanbonro 321, Gunpo, Gyeonggi-do 15-865, Republic of Korea

## Abstract

*Introduction*. Fluid-attenuated inversion recovery hyperintense vessels (FHVs) can be seen in patients with occlusion or severe stenosis of the cerebral arteries. FHVs are known to reflect stagnant or slow blood flow within the cerebral artery.* Case Report*. A 75-year-old woman presented with suddenly developed gait disturbance. She had a history of hypertension, heart failure, and dementia. Brain MRI demonstrated FHVs within both middle cerebral arteries (MCAs). However, there was no acute ischemic lesion and severe stenosis or occlusion of the cerebral arteries. In the baseline routine laboratory investigations, the AST, ALT, and B-type natriuretic peptide levels were elevated. Transthoracic echocardiography (TTE) showed mitral valve prolapse with severe regurgitation. Blood pressure control and conservative management for ischemic hepatitis were performed. After 7 days, the transaminase levels were normalized, and the patient was able to walk with normal gait.* Conclusions*. In this patient, underlying chronic cerebral hypoperfusion and additionally decreased systemic perfusion seemed to provoke ischemic hepatitis and contribute to the development of FHVs.

## 1. Introduction

Fluid-attenuated inversion recovery hyperintense vessels (FHVs) can be seen in ischemic stroke patients with arterial occlusion or significant stenosis and in patients with cerebral arterial occlusion but without infarction, such as those with moyamoya disease [1, 2]. The mechanism of FHVs is known to be related to slow or stagnant blood flow [1]. We report a patient with FHVs reflecting slow intracranial blood flow due to severe heart failure.

## 2. Case Report

**Figure 1:**
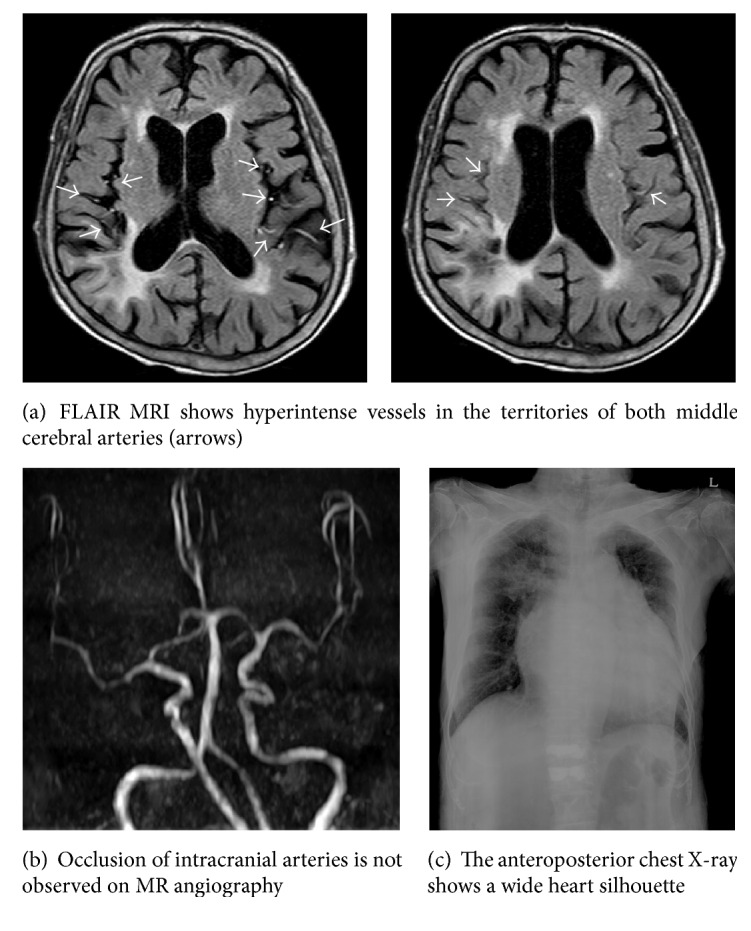
Magnetic resonance and chest X-ray images.

After approval from the Institutional Review Board of our institute, we retrospectively reviewed the medical records of a patient. A 75-year-old woman presented with gait disturbance with general weakness. She had a history of hypertension and heart failure. Additionally, she was diagnosed with dementia 4 years ago and was taking donepezil. Although she was demented, her activities of daily living (ADL) were preserved. There were no focal neurologic deficits. MRI images were obtained with a 1.5-T scanner (Philips Intera, Philips Medical Systems, Best, Netherlands). The fluid-attenuated inversion recovery (FLAIR) parameters for the scanner were set as follows: repetition time/echo time (TR/TE) = 9000/125 ms, inversion time (TI) = 2500 ms, field of view (FOV) = 230 × 230 mm, matrix size = 288 × 190, slice thickness = 5 mm, and interslice gap = 2 mm. Time-of-flight (TOF) MR angiography (MRA) was obtained using the following parameters: TR/TE = 25/6.9 ms, flip angle = 20 degrees, FOV = 158 × 158 mm, matrix size = 380 × 244, and slice thickness = 0.9 mm. Brain MRI demonstrated FHVs within both middle cerebral arteries (MCAs) (Figure 1(a)). Additionally, brain MRI demonstrated leukoaraiosis and cerebral atrophy. Acute ischemic lesion was not detected on diffusion MRI, and severe stenotic or occlusive lesion was also not detected on TOF-MRA (Figure 1(b)). The initial blood pressure was 95/55 mmHg. In the baseline routine laboratory investigations, aspartate transaminase (AST) and alanine transaminase (ALT) levels were markedly elevated (AST = 1672 U/L, ALT = 1404 U/L). The B-type natriuretic peptide level was elevated moderately (529.45 pg/mL). She was seronegative for HBV and HCV and was not on medications known to have hepatotoxicity. A chest X-ray showed a wide heart silhouette (Figure 1(c)). Transthoracic echocardiography (TTE) revealed that the prolapse of the anterior mitral valve had led to severe regurgitation of the mitral and tricuspid valves (see Supplementary video 1 in Supplementary Material available online at http://dx.doi.org/10.1155/2016/5169056). Blood pressure control and conservative management for ischemic hepatitis were conducted. Intravenous administration of dopamine was performed for the augmentation of hepatic blood flow. The daily dose of oral spironolactone was reduced from 50 mg to 12.5 mg, and the dose of carvedilol was reduced from 12.5 mg to 3.125 mg. The patient was able to walk as her general condition improved. In the next 7 days, her transaminase levels were decreased and normalized. She was discharged after two weeks of admission with an ambulatory status.

## 3. Discussion

This case is the first report of FHVs in a patient without intra- or extracranial arterial occlusive diseases. Even if acute ischemic stroke is caused by an occlusion of a cerebral artery, FHVs are not detected when the cerebral blood flow velocity is fast through the collateral circulation [3]. With regard to the clinical meaning of FHVs, distal FHVs reflect the collateral circulation, and the outcome is favorable for distal FHVs in acute ischemic stroke patients [4]. Conversely, FHVs are associated with poor outcomes 3 months after thrombolysis in acute ischemic stroke [5]. FHVs, called the “ivy sign” in moyamoya disease, are known to reflect decreased cerebral blood flow and are related to a severe degree of cerebral ischemia [6].

Cerebral blood flow is decreased in patients with heart failure [7]. About 35–50% of patients with heart failure have cognitive dysfunction [8]. Patients with vascular dementia also have significantly lower cerebral blood flow velocity compared with healthy age-matched controls [9]. The major cause of cognitive impairment in patients with advanced heart failure is cerebral hypoperfusion [10]. Decreases of cerebral perfusion have been linked to the development of white matter hyperintensity [11]. White matter hyperintensity lesions on FLAIR MRI were seen in this patient with dementia. It is possible that chronic cerebral hypoperfusion is one of the causes of dementia in this patient. Reduced systemic perfusion that led to acute ischemic hepatitis might have affected blood flow to the brain. Reduced cerebral blood flow with comorbid conditions seemed to have contributed to the development of FHVs in this patient. Decreased cerebral blood flow in patients with heart failure is related to mitral regurgitation grade and tricuspid regurgitation and to pulmonary hypertension, but not to left ventricular ejection fraction (LVEF) [8].

The presenting case has a single limitation. Underlying intracranial stenosis of both MCAs from TOF MRA could not be excluded in this patient due to poor image quality. Although transfemoral cerebral angiography was needed to evaluate the degree of stenosis accurately, it was not performed in this patient. However, TOF MRA has a tendency to overestimate the degree of intracranial stenosis [12], and FHVs were only detected in severely stenotic intracranial arteries with more than 90% stenosis [13]. Therefore, even though intracranial stenosis was present in this patient, it may not be a significant factor resulting in FHVs.

Further clinical study and review of imaging data on heart failure patients with comorbidity are needed to identify similar findings and to investigate the correlation of cerebral hypoperfusion with FHVs.

## Supplementary Material

Trans-thoracic echocardiography showed severe left atrial enlargement and severe mitral regurgitation.

## References

[1] Kamran S., Bates V., Bakshi R., Wright P., Kinkel W., Miletich R. (2000). Significance of hyperintense vessels on FLAIR MRI in acute stroke. *Neurology*.

[2] Maeda M., Tsuchida C. (1999). ‘Ivy sign’ on fluid-attenuated inversion-recovery images in childhood moyamoya disease. *American Journal of Neuroradiology*.

[3] Lee S. H., Seo K. D., Kim J. H., Suh S. H., Ahn S. J., Lee K.-Y. (2016). Correlation between hyperintense vessels on FLAIR imaging and arterial circulation time on cerebral angiography. *Magnetic Resonance in Medical Sciences*.

[4] Lee K. Y., Latour L. L., Luby M., Hsia A. W., Merino J. G., Warach M. S. (2009). Distal hyperintense vessels on FLAIR: an MRI marker for collateral circulation in acute stroke?. *Neurology*.

[5] Ebinger M., Kufner A., Galinovic I. (2012). Fluid-attenuated inversion recovery images and stroke outcome after thrombolysis. *Stroke*.

[6] Mori N., Mugikura S., Higano S. (2009). The leptomeningeal ‘ivy sign’ on fluid-attenuated inversion recovery MR imaging in Moyamoya disease: a sign of decreased cerebral vascular reserve?. *American Journal of Neuroradiology*.

[7] Gruhn N., Larsen F. S., Boesgaard S. (2001). Cerebral blood flow in patients with chronic heart failure before and after heart transplantation. *Stroke*.

[8] Choi B.-R., Kim J. S., Yang Y. J. (2006). Factors associated with decreased cerebral blood flow in congestive heart failure secondary to idiopathic dilated cardiomyopathy. *The American Journal of Cardiology*.

[9] Sabayan B., Jansen S., Oleksik A. M. (2012). Cerebrovascular hemodynamics in Alzheimer's disease and vascular dementia: a meta-analysis of transcranial Doppler studies. *Ageing Research Reviews*.

[10] Román G. C. (2004). Brain hypoperfusion: a critical factor in vascular dementia. *Neurological Research*.

[11] Vogels R. L. C., Scheltens P., Schroeder-Tanka J. M., Weinstein H. C. (2007). Cognitive impairment in heart failure: a systematic review of the literature. *European Journal of Heart Failure*.

[12] Choi C. G., Lee D. H., Lee J. H. (2007). Detection of intracranial atherosclerotic steno-occlusive disease with 3D time-of-flight magnetic resonance angiography with sensitivity encoding at 3T. *American Journal of Neuroradiology*.

[13] Liu W., Xu G., Yue X. (2011). Hyperintense vessels on Flair: a useful non-invasive method for assessing intracerebral collaterals. *European Journal of Radiology*.

